# Impact of Bt Brinjal Cultivation in the Market Value Chain in Five Districts of Bangladesh

**DOI:** 10.3389/fbioe.2020.00498

**Published:** 2020-05-25

**Authors:** Anthony M. Shelton, Sayed H. Sarwer, Md. J. Hossain, Graham Brookes, Vijay Paranjape

**Affiliations:** ^1^Department of Entomology, Cornell AgriTech, New York State Agricultural Experiment Station, Geneva, NY, United States; ^2^Feed the Future South Asia Eggplant Improvement Partnership, Cornell University, Ithaca, NY, United States; ^3^PG Economics, Stafford House, Dorchester, United Kingdom; ^4^Sathguru Management Consultants Pvt Ltd, Hyderabad, India

**Keywords:** brinjal, eggplant, Bt, Bangladesh, value

## Abstract

Eggplant (brinjal) is a popular vegetable that provides an important source of income for small, resource-poor Bangladeshi farmers. The biggest constraint to brinjal production is the eggplant fruit and shoot borer (EFSB). This study was conducted in 2019 in five districts in Bangladesh and examined the impacts of using genetically engineered, insect-resistant brinjal (Bt brinjal) on its value and marketing. Based on a survey of Bt and non-Bt farmers, results indicate that Bt brinjal provided an average of 19.6% higher yield and 21.7% higher revenue compared to non-Bt varieties. On a per tonne basis, the revenue benefit of using Bt brinjal was 1.7%, reflecting different levels of acceptability among trade buyers and consumers. Some were prepared to pay higher prices for Bt brinjal compared to non-Bt brinjal because the fruit was less damaged, while others paid a price discount because the Bt brinjal was not available in preferred local varieties. Labor use, expressed in 8-h days, for harvesting, grading, and packaging of Bt brinjal was 14% higher for Bt brinjal, reflecting the increased yields of Bt brinjal. 83.1% of Bt brinjal growers were satisfied with the yields obtained, and 80.6% were satisfied with the quality of fruit. This contrasts with non-Bt brinjal growers where 58.7% were satisfied with their yields and 28% indicated that a large portion of their fruit was infested, not a concern for Bt brinjal. Three-quarters of Bt brinjal growers planned to plant Bt brinjal next season because of the apparent benefits achieved of higher yields, revenue and fruit quality. Many also highlighted the benefits of reduced insecticides. Of the non-Bt growers, 39.6% had not heard of Bt brinjal. However, after hearing more about the impact of the technology, 71.4% of them indicated they planned to grow Bt brinjal next season. These findings suggest there are significant benefits of Bt brinjal and highlight the importance of making the technology available in more varieties that are suitable to local conditions and consumer preferences. Additional studies are warranted to corroborate these findings and explore in more detail the factors influencing decisions made by farmers and consumers regarding Bt brinjal.

## Introduction

Brinjal, or eggplant (*Solanum melongena* L.), is the second most important vegetable grown in Bangladesh, by about 150,000 resource-poor farmers on 50,955 hectares with a total production of 507,000 metric tonnes in 2018 (Bangladesh Bureau of Statistics (BBS), 2018). Brinjal accounted for 4.7 and 9.6%, respectively, of all winter and summer vegetable production in 2018 (Bangladesh Bureau of Statistics (BBS), 2018). Brinjal is grown in almost all agro-climatic zones with over 100 different varieties under cultivation, offering fruits of different color, size, shape, and taste. Brinjal is seriously affected by insect infestations, primarily the eggplant fruit and shoot borer (EFSB), *Leucinodes orbonalis* Guenée (Lepidoptera: Crambidae). EFSB causes between 30 and 60% yield loss, even when the crop is frequently sprayed with insecticides (Mondal and Akter, 2018). EFSB larvae damage the eggplant shoots and flowers, although the most serious damage is caused by their boring into the fruit and rendering it unmarketable. Brinjal crops are typically sprayed with insecticides over 80 times in a season of 4–5 months in all of the major growing areas in Bangladesh (Meherunnahar and Paul, 2009). This frequent application of insecticides results in very high pesticide residue levels on the fruit, kills beneficial insects, exposes farm workers to hazards, and contributes to polluting the local environment (Rahman, 2013).

Genetically engineered, insect resistant brinjal with the *cry1Ac* gene (Bt brinjal) was developed by the India-based Maharashtra Hybrid Seed Company (Mahyco) to provide an effective control of EFSB. The Agricultural Biotechnology Support Project II at Cornell University, supported by the United States Agency for International Development, facilitated the transfer of the Bt brinjal event (“EE-1”) to the Bangladesh Agricultural Research Institute (BARI) and this event was introgressed into several local and commercially popular open-pollinated brinjal varieties (Shelton et al., 2018). The resulting nine Bt varieties underwent 7 years of greenhouse and confined field trials by BARI in various geographic locations in Bangladesh to test their efficacy and environmental safety. Out of those nine Bt varieties, four were subsequently approved for cultivation by the National Committee on Biosafety (NCB) of Bangladesh on October 2013. The released Bt varieties are BARI Bt Begun-1, BARI Bt Begun-2, BARI Bt Begun-3, and BARI Bt Begun-4 which are Bt isolines of Uttara, Nayantara, Kazla, and ISD006, respectively (Shelton et al., 2018). In this report they are referred to as Bt brinjal-1, Bt brinjal-2, Bt brinjal-4, and Bt brinjal-4.

These four Bt varieties are open-pollinated, which allows farmers to save seed for re-use. However, farmers are discouraged from using saved seed for multiple seasons because of potential outcrossing to other varieties, especially to non-Bt brinjal that are used in border rows as part of a refuge in a resistance management strategy (Shelton et al., 2019). After approval, the government supplied Bt brinjal seedlings to 20 selected farmers in four districts for cultivation in 2014, entrusting BARI personnel to provide training, guidance, and supervision on crop management to farmers. Since 2014, the adoption of Bt brinjal has been rapid (Table 1). Farmers now obtain their seed from three different Bangladeshi organizations: BARI, Department of Agricultural Extension (DAE) and the Bangladesh Agricultural Development Corporation (BADC) with seed distributed for free, except for a small charge (< US $0.10 per gram, equal to 8 Bangladesh Taka (BDT) local currency) if sourced from BADC. In 2018–9, Bt brinjal was grown by 20,695 farmers on 1,213.3 ha, equal to nearly 2.5% of the total crop (Table 1).

**Table 1:** Farmer adoption of Bt brinjal in Bangladesh by source of seed: BARI (Bangladesh Agricultural Research Institute); DAE (Department of Agricultural Extension); BADC (Bangladesh Agricultural Development Corporation). Figures do not include farmer-saved seed.

**Year**	**Number of farmers**				**Area in production (ha)**			
	BARI	DAE	BADC	Total	BARI	DAE	BADC	Total
2013–14	20	0	0	20	2.83	0	0	2.8
2014–15	108	0	0	108	14.6	0	0	14.6
2015–16	250	0	0	250	10.1	0	0	10.1
2016–17	512	6,000	0	6,512	20.6	485.6	0	506.3
2017–18	581	7,601	19,430	27,612	38.9	567.8	786.3	1,392.9
2018–19	225	7,070	13,400	20,695	15.0	656.0	542.3	1,213.3

*Source: USAID Feed the Future South Asia Eggplant Improvement Partnership Project, 2019*.

Several studies have documented the performance of Bt brinjal. In a study conducted by BARI scientists in 35 districts during the 2016–17 cropping season with 505 Bt brinjal farmers and 350 non-Bt brinjal farmers, net returns/ha, were US$2,151/ha for Bt brinjal as compared to US$357/ha for non-Bt brinjal, a 6-fold difference (Rashid et al., 2018). This study also identified that farmers spent 61% less on pesticides compared to non-Bt brinjal farmers and experienced no yield losses due to the EFSB. In a 2-year study conducted by Prodhan et al. (2018), all four Bt brinjal varieties provided virtually complete control of EFSB without the use of insecticides for EFSB control, and had higher gross returns than their non-Bt equivalents. A report by Ahmed et al. (2019) evaluated the impacts of the Bt brinjal technology on production systems, marketability, and human health. The study compared results of 600 Bt brinjal farmers and 600 non-Bt brinjal farmers living in 200 villages in four districts in the northwest of Bangladesh during the winter season of 2017-18. The results demonstrated that Bt brinjal farmers experienced significantly lower pesticide use, a reduction in overall production costs, increased yields, and provided higher profits. However, the study only included one of the four commercialized Bt varieties.

The overall objective of the present study was to identify the impact of using the four Bt brinjal varieties on the market value of the crop relative to the market value of conventional, non-Bt brinjal varieties. The specific objectives were: (1) assess the impact on the revenue generation in the value chain; (2) assess the labor use impact; (3) identify preferences and perceptions toward Bt brinjal among the farmers, traders, and consumers.

## Materials and Methods

### Study Areas

Five important brinjal producing districts in Bangladesh were selected: Rangpur, Bogra, Rajshahi, Jessore, and Tangail. Within each district, one upazila (subdistrict) was randomly selected for farmer interviews, resulting in a study area of five upazilas across the five districts.

### Sample Size

In each upazila, subsets of Bt brinjal and non*-*Bt brinjal farmers were randomly selected. The original plan was to collect data from a total of 500 farms, 250 Bt, and 250 conventional farms. However, after discarding incomplete survey responses, the total numbers of useable interview responses were 195 Bt farmers and 196 non-Bt farmers. Farmers chose to grow either a Bt brinjal variety or a non-Bt brinjal variety on their own.

### Data Collection and Presentation

Face to face interviews were conducted between February and May 2019, following predesigned and pretested questionnaires. Each set of questionnaires was divided into three parts:

Part 1: General information about each farmer's enterprise that was collected before harvesting.

Part II: Information about harvesting and marketing of brinjal that was collected during harvesting and the subsequent marketing period.

Part III: Post-harvest qualitative views on perceptions of Bt and non-Bt brinjal were collected after completion of harvesting and marketing.

Data are presented as mean values without deeper statistical analysis. The sample sizes and complexity of factors involved limited more detailed analysis, but the means are indicative of trends that can be followed up with more detailed studies.

Data on the monetary value are presented in local currency, the Bangladesh Taka (BDT) where 1$US equals 84 BDT.

## Results

### Quantitative Impacts Collected Before Harvesting

In terms of age and sex distribution, primary and secondary occupation, average household size and average area of cultivated land, Bt brinjal farmers appeared not notably different from non-Bt farmers. However, Bt farmers owned 9% more land (0.83 vs. 0.76 ha) and had an 8% higher overall annual farm income (BDT 192,190 vs. 177,406). The land devoted to Bt and non-Bt brinjal cultivation varied by district (Table 2). Over all districts, the survey revealed slightly larger fields grown to non-Bt brinjal compared to Bt brinjal (0.08 vs. 0.07 ha). Bt farmers obtained advice to grow Bt brinjal primarily from BARI (63.3%) and DAE (33.3%). Non-Bt brinjal farmers used their traditional knowledge of brinjal production.

**Table 2:** Average field size in hectares (ha) by district under Bt and non-Bt brinjal cultivation in the survey, 2019.

**District**	**Jessore**		**Tangail**		**Bogra**		**Rajshahi**		**Rangpur**		**All Districts**	
	**Mean**	***N***	**Mean**	***N***	**Mean**	***N***	**Mean**	***N***	**Mean**	***N***	**Mean**	***N***
Bt brinjal	0.06	33	0.09	40	0.07	50	0.07	37	0.05	35	0.07	195
Non-Bt brinjal	0.11	32	0.11	39	0.07	50	0.08	38	0.05	37	0.08	196

### Quantitative Impacts Collected During Harvesting and Marketing Periods

#### Harvesting and Yield of Brinjal

Brinjal enters the marketing chain immediately after harvesting with farmers generally harvesting fruits 2–3 times a week during the harvesting season. The survey identified that the total number of harvests ranged from 24 to 32, occurring twice a week during the peak production period. Local traders commonly visit farmers' fields to buy fruit in bulk which they then sell at local markets either to the large wholesale traders or direct to consumers. The larger wholesale traders also procure fruits from farmers directly if they visit local markets. They then sell the brinjal at wholesale markets in the cities but this requires transportation and results in a time lag of 6–12 h before sale of the fruits in these urban markets.

The average number of harvests of fruits was the same (27.4) for both Bt and non-Bt brinjal farmers, though there was some variation between districts (Table 3). Overall, cultivation of Bt brinjal had no apparent impact on the frequency and number of harvests.

**Table 3:** Average number of harvests and yield per hectare by district for Bt and non-Bt brinjal, 2019.

**District**	**No. of Harvests**		**Yield per hectare (1,000 kg)**		**Yield difference (%)**
	**Bt brinjal**	**Non-Bt brinjal**	**Bt brinjal**	**Non-Bt brinjal**	
Jessore	26.8	27.6	18.35	16.02	14.5
Tangail	25.0	27.8	18.12	15.69	15.5
Bogra	27.4	25.3	21.23	17.28	22.9
Rajshahi	28.7	27.8	20.91	17.27	21.1
Rangpur	29.3	29.3	20.17	17.47	15.4
All Districts	27.4	27.4	19.80	16.55	19.6

The average yield of Bt brinjal varieties/ha was 19.8 tonnes compared to 16.55 tonnes/ha for non-Bt brinjal varieties, indicating a 19.6% higher yield of Bt brinjal. The highest yield difference (+22.9%) was observed in Bogra, with the lowest yield difference in Jessore (+ 14.5%).

#### Selection of Varieties and Their Yield per Hectare

In each district, farmers typically plant varieties most suited to the local conditions and markets. Some of the Bt varieties differed from the preferred local varieties. Hence, preferences varied by district. Bt brinjal varieties 4, 3, and 2 were grown in Jessore, varieties 3 and 2 were grown in Tangail, varieties 4, 3, and 1 were grown in Bogra, varieties 4, 3, 2, and 1 were grown in Rangpur and only variety 4 was grown in Rajshahi district (Table 4). Our data suggest there were large differences in the yield/ha of the same Bt variety across districts. For example, the yield of Bt brinjal 4 was 17.6 tonnes/ha in Jessore, whereas it was 23.3 tonnes/ha in Bogra, 20.9 tonnes/ha in Rajshahi, and 20.3 tonnes/ha in Rangpur. Similarly, the average yield of Bt brinjal 3 was 20.8 tonnes/ha in Jessore, while it was only 17.5 tonnes/ha in Tangail. These yield differences suggest that all of the Bt varieties were not equally suitable for local growing conditions or commercially attractive enough to farmers in each district.

**Table 4:** Average yield (1,000 kg per ha) of Bt brinjal varieties by district, 2019.

**Bt brinjal varieties**	**Jessore**	**Tangail**	**Bogra**	**Rajshahi**	**Rangpur**	**All Districts**
BARI Bt brinjal-4	17.6		23.3	20.9	20.3	20.7
BARI Bt brinjal-3	20.8	17.5	21.1		19.5	18.2
BARI Bt brinjal-2	20.1	18.5			21.8	18.7
BARI Bt brinjal-1			19.6		19.2	19.5
All Bt varieties	18.4	18.1	21.2	20.9	20.2	19.8

#### Average Gross Revenue Per Hectare

Within the study area, our data suggest that Bt brinjal varieties always earned higher revenue/ha than the non-Bt brinjal varieties (Table 5). The average gross revenue after selling Bt brinjal was estimated at BDT 312,478/ha (about $US 3,720) compared to BDT 256,718/ha (about $US 3,056) for non-Bt brinjal, a 21.7% higher revenue for the Bt varieties. The highest revenue increase (+30.2%) was observed in the Rajshahi district, while the lowest (+15.3%) increase was observed in Tangail. In terms of returns/tonne of brinjal, the average revenue for Bt brinjal was BDT 15,769/tonne, or 1.7% higher than the revenue for non-Bt brinjal.

**Table 5:** Average gross revenue for Bt and non-Bt brinjal in BDT^*^, 2019.

**Districts**	**Per hectare**		**Per 1,000 kg**		**Revenue Increase (%)**	
	**Bt brinjal**	**Non-Bt brinjal**	**Bt brinjal**	**Non-Bt brinjal**	**Per hectare**	**Per 1,000 kg**
Jessore	294,985	255,701	16,073	15,960	15.4	0.7
Tangail	285,500	247,540	15,753	15,771	15.3	−0.1
Bogra	335,941	263,627	15,827	15,257	27.4	3.7
Rajshahi	344,535	264,663	16,474	15,323	30.2	7.5
Rangpur	306,645	254,519	15,201	14,570	20.5	4.3
All Districts	312,478	256,718	15,769	15,510	21.7	1.7

* *US$1 = BDT 84*.

Differences in revenue/tonne between Bt and non-Bt brinjals varied widely across the districts. In Rajshahi, Bt brinjal earned 7.5% higher revenue than non-Bt brinjal, while the revenue/tonne advantage of Bt brinjal was only 0.7% higher in Jessore and 0.1% lower in Tangail. This difference appeared to be due to the wide price difference between Bt and non-Bt brinjal in Jessore and Tangail and reflects differences in the acceptability of the available Bt brinjal varieties vs. traditional non-Bt brinjal varieties to buyers, traders, and consumers. The level of acceptability among buyers of the Bt varieties appeared to be much higher in Rajshahi, Bogra, and Rangpur compared to Jessore and Tangail where buyers seemed to prefer the local (non-Bt) varieties and hence were prepared to pay a higher price than for the Bt brinjal.

#### Price, Utilization, and Revenue at Different Levels of the Market

The price of brinjal varies by the nature of the market in which it is sold. For the purpose of this study, home-consumed brinjal was assumed to be traded at the same price offered by local traders at farmers' fields. Unsold produce that remained after the end of formal sales in local markets was valued at zero if fed to cows, or assumed to be sold in lots to local traders/consumers at half of the market price. Therefore, the average value (price) attributed to the unsold element of produce (see Table 6) was weighted according to the volumes sold in lots at the end of market days (at 50% of the market price) and the volume fed to cows (assumed to have no value).

**Table 6:** How the product was segmented in the market, its price and the revenue generated for Bt and non-Bt brinjal at different levels of the market, 2019.

**How product was segmented based on percentage of the total product**		
**Level of Market**	**Bt brinjal (%)**	**Non-Bt brinjal (%)**
Home consumption	1.7	2.3
On-farm sales to traders	16.3	6.7
Wholesale at local market	58.2	74.6
Retail sale at local market	20.2	7.7
Unsold product (disposal)	3.6	8.7
Total	100.0	100.0
Price in BDT^*^		
	**Bt brinjal per kg**	**Non-Bt brinjal per kg**
Home consumption	14.48	14.65
On-farm wholesale	14.48	14.65
Wholesale at local market	16.43	16.09
Retail sale at local market	16.94	15.29
Unsold product (disposal)	7.90	7.27
All	15.78	15.51
**Revenue generated by selling in percentage of the total value of fruits**		
**Level of Market**	**Bt brinjal (%)**	**Non-Bt brinjal (%)**
Home consumption	1.6	2.2
On-farm sales to traders	15.0	6.3
Wholesale at local market	60.5	78.4
Retail sales at local market	21.7	8.5
Unsold product (disposal)	1.8	4.6
Total	100.0	100.0

* *US$1 = BDT 84*.

The data suggest the prices of Bt brinjal sold at the local markets either to wholesalers or direct to consumers (retail sales) were higher than the average price of non-Bt brinjal, although for on-farm sales to wholesalers, the price paid for non-Bt brinjal was slightly higher (Table 6). The average price of Bt brinjal across all markets and uses was BDT 15.78/kg compared to BDT 15.51/kg for non-Bt brinjal.

The majority of all harvested fruits was sold in the local markets via wholesales to traders. Nearly three-quarters (74.6%) of the non-Bt brinjal fruit was sold in this way, compared to about 58.2% of the Bt brinjal. The next most important outlets for Bt brinjal were retail sales to end consumers (20.2% of sales) and on-farm sales to traders (16.3% of sales). In contrast, only 6.7% of non-Bt brinjal sales were on-farm sales to traders and only 7.7% of Bt brinjal sales were to end-users (retail sales). Home consumption levels of fruit were similar for both types of fruit (about 2%), although the level of home consumption was slightly higher (0.05%) for non-Bt farmers. The unsold proportion of marketed brinjal (used as animal feed or sold off at the end of the market day) was higher for non-Bt brinjal (8.7% of sales/uses) compared to the 3.6% for Bt brinjal. These data suggest that local traders and consumers preferred Bt brinjal to non-Bt brinjal, presumably because the fruits were less damaged by the EFSB.

Largely reflecting the proportion of brinjal sold in different markets, the total revenue earned from the sale of brinjal to wholesales in local markets was highest, with 60.5% of all Bt brinjal revenue coming from this sales channel and 78.4% of the non-Bt brinjal revenue coming from this sales channel. The next most important sales channels, in terms of revenue generation for Bt brinjal farmers, were retail sales to consumers which accounted for 21.7% of total revenue and on-farm sales to traders which generated 15% of total revenue. This contrasts with the non-Bt brinjal, where these two sales channels were responsible for much smaller shares of total revenue generation (8.5% for retail sales and 6.3% for on-farm sales to traders). These data suggest that while local traders preferred Bt brinjal to non-Bt brinjal for selling in local markets, non-Bt brinjals were the preferred product for traders selling in city markets. This preference for non-Bt brinjal for sale in city markets apparently was due to the non-Bt brinjal being better able to retain its skin color and texture than the Bt brinjal after 6–12 h of transportation time to city wholesale markets. Such skin color and texture are a reflection of the variety and not whether it is Bt or non-Bt.

#### Labor and Wages for Harvesting, Grading, and Packaging

The data suggest there was a notable employment impact associated with Bt brinjal production due to the increased yield of the marketable product (Table 7). Across all districts, the labor required/ha for harvesting, grading and packaging of Bt brinjal was estimated at 113.1 days (8-h day) compared to 99 days for non-Bt brinjal. An additional 21.8 days/ha were required for Bt brinjal farmers in the Jessore district compared to the non-Bt brinjal farmers. In contrast, in Tangail Bt farmers employed 1.8 fewer days for these activities than the non-Bt growers.

Table 7Labor and wages for harvesting, grading, and packaging Bt and non-Bt brinjal by district, 2019.

**Table:** 

**Labor in 8-hour days**						
**Districts**	**Bt brinjal (days)**			**Non-Bt brinjal (days)**		
	**Family**	**Hired**	**Total**	**Family**	**Hired**	**Total**
Jessore	75.6	46.2	121.8	57.6	42.5	100.0
Tangail	59.0	41.8	100.8	57.8	44.8	102.6
Bogra	67.2	46.2	113.4	67.2	43.9	111.1
Rajshahi	73.8	44.4	118.2	59.0	37.0	95.9
Rangpur	71.5	44.7	116.2	68.6	44.1	112.7
All Districts	68.4	44.7	113.1	59.2	39.8	99.0

**Table:** 

Wages paid for harvesting, grading and packaging in BDT^*^				
**Districts**	**Wage per hectare**		**Wage per 1,000 kg**	
	**Bt brinjal**	**Non-Bt brinjal**	**Bt brinjal**	**Non-Bt brinjal**
Jessore	17,897	16,459	967	1,027
Tangail	16,844	16,958	929	1,081
Bogra	18,655	17,277	879	1,000
Rajshahi	18,699	17,719	934	1,026
Rangpur	16,222	16,517	799	946
All Districts	17,829	17,099	918	1,033

* *US$1 = BDT 84*.

Harvesting, grading, and packaging are most commonly done in the early part of the day and completed in a few hours (not requiring a whole 8-h working day). This makes such work more suitable for family labor than needing to hire external labor. Out of the 14.1 full days of additional labor/ha used by Bt brinjal farmers compared to non-Bt farmers, 9.2 days were family labor and 4.9 were hired labor (60 and 40%, respectively).

Total wages paid to hired labor for harvesting, grading, and packaging were BDT 17,829/ha for Bt brinjal as compared to BDT 17,099 for non-Bt brinjal. However, in terms of harvesting labor costs/tonne of produce, the hired labor cost was lower for Bt brinjal farmers (BDT 918) compared to non-Bt brinjal farmers (BDT 1033).

#### Marketing Costs

Marketing costs/ha, including costs of transportation from farm to market and market tolls, appeared to be higher for Bt brinjal (BDT 23,677/ha of crop) compared to BDT 19,826 for non-Bt brinjal (Table 8). However, the marketing cost of Bt brinjal/tonne was similar to the cost of non-Bt brinjal. These differences appear to reflect the yield differences between the two crops.

**Table 8:** Marketing cost for Bt and non-Bt brinjal by district in BDT^*^, 2019.

**District**	**Cost per hectare**		**Cost per 1,000 kg**	
	**Bt brinjal**	**Non-Bt brinjal**	**Bt brinjal**	**Non-Bt brinjal**
Jessore	24,599	20,593	1,287	1,285
Tangail	22,360	18,213	1,219	1,179
Bogra	24,129	20,858	1,213	1,207
Rajshahi	21,692	18,741	1,117	1,085
Rangpur	26,025	21,684	1,305	1,241
All Districts	23,677	19,826	1,227	1,203

* *US$1 = BDT 84*.

#### Qualitative Impacts Collected After Completion of Harvesting and Marketing

In the survey, additional questions were asked of the farmers about their current knowledge and experience with the Bt brinjal relative to local non-Bt brinjal varieties in order to assess reasons for adoption and prospects for future use of the technology.

#### Overall Satisfaction With the Bt Brinjal

Over all districts, 80.6% of the Bt brinjal farmers, compared to 71.9% of non-Bt brinjal farmers, appeared to be satisfied with the quality of their respective produce (Table 9). A larger proportion of Bt farmers (83.1%) were satisfied with the fruit yield/ha compared to yield satisfaction levels of non-Bt farmers (58.7%). Only in Jessore and Tangail were there any farmers expressing less satisfaction with Bt brinjal than non-Bt brinjal.

**Table 9:** Grower satisfaction with the quality of fruit and yield of Bt and non-Bt brinjal by district, 2019.

**Districts**	**% satisfied with quality**		**% satisfied with yield**	
	**Bt brinjal**	**Non-Bt brinjal**	**Bt brinjal**	**Non-Bt brinjal**
Jessore	66.7	84.4	53.5	81.3
Tangail	54.5	100.0	65.0	87.2
Bogra	96.0	40.0	98.0	46.0
Rajshahi	91.9	73.7	97.3	26.3
Rangpur	95.0	73.0	100.0	59.6
All Districts	80.6	71.9	83.1	58.7

*Figures represent the percentage of the total number of farmers*.

#### Problems Encountered With Growing, Selling, or Marketing Produce

For the 19.4% of Bt brinjal growers that expressed some concern with their crop, the main concern related to skin of the brinjal (14.9%) that adversely affected the product quality during transportation from local markets to city markets (Table 10). In relation to concerns with the quality of non-Bt produce, of the 28.1% of farmers who expressed some concern, the main concern was insect infestation in a large portion of their harvested fruits (26.5%).

Table 10Grower concerns with growing, selling, and marketing Bt brinjal and non-Bt brinjal by district, 2019.

**Table:** 

**Growing**						
**Quality concerns**	**Jessore**	**Tangail**	**Bogra**	**Rajshahi**	**Rangpur**	**All Districts**
**Bt brinjal**						
Color, shape and size of fruit not attractive	18.2	20.0	0	0	0	7.2
Tough fruit	0	10.0	4.0	0	0	3.1
Skin affected during transportation	30.3	35.0	0	8.2	5.0	14.9
No comments	9.1	2.5	0	0	0	2.1
*Farmers expressing concerns (%)*	33.3	45.5	4.0	8.1	5.0	19.4
**Non-Bt brinjal**						
A large portion of the harvest was infested	15.6	0	54.0	26.3	27.0	26.5
*Farmers expressing concerns (%)*	15.6	0	60.0	26.3	27.0	28.1

**Table:** 

**Selling**				
**Districts**	**Bt brinjal**		**Non- Bt brinjal**	
	**Faced problems**	**Did not face problems**	**Faced Problems**	**Did not face problems**
Jessore	93.9	6.1	34.4	65.6
Tangail	80.0	20.0	30.8	69.2
Bogra	22.0	78.0	48.0	52.0
Rajshahi	16.2	83.8	73.7	26.3
Rangpur	29.1	70.9	54.1	45.9
All Districts	56.9	43.1	48.5	51.5

**Table:** 

**Marketing**						
**Problems**	**Jessore**	**Tangail**	**Bogra**	**Rajshahi**	**Rangpur**	**All Districts**
**Bt brinjal**						
Price of Bt brinjal was lower than non- Bt brinjal	93.9	80.0	10.0	16.2	0.0	37.9
Traders are less interested to buy Bt brinjal	84.8	72.5	12.0	16.2	8.6	36.9
Consumers are less interested to buy	81.8	42.5	12.0	16.2	0	28.7
Traders/consumers had negative perception	54.5	42.5	0	2.7	8.6	20.0
Color and shape was not like the local brinjal	9.1	15.0	0	0	0	4.6
Farmers facing problems (%)	93.9	80.0	22.0	16.2	29.1	56.9
**Non-Bt brinjal**						
Did not get expected price as the fruits were infested	18.8	30.8	48.0	73.7	54.1	45.9
A large amount remained unsold	3.1	0	12.0	2.6	0	4.1
Farmers facing problems (%)	34.4	30.8	48.0	73.7	54.1	48.5

*Figures represent the percentage of farmers with concerns*.

Overall, 56.9% of Bt farmers and 48.5% of non-Bt farmers indicated that they had faced problems selling their produce. The most popular complaint of the Bt brinjal farmers who had experienced problems was that the price received was lower than the price of local and popular non-Bt varieties (37.9%), although this perception is inconsistent with the actual gross revenue data presented in Table 5.

Overall, 36.9% of the Bt brinjal growers perceived that traders were not interested in buying Bt brinjal and 28.7% of these farmers also perceived that consumers were not interested in buying Bt brinjal. As highlighted above relating to perceptions relating to difficulties selling Bt brinjal, these perceptions about trader and consumer purchasing preferences appeared to be inconsistent with the volumes left unsold (see below).

#### Outcome of Marketing Bt and Non-Bt Brinjal

Of the Bt brinjal farmers, there was considerable variation between districts but, over all districts, 21.0% reported that a large portion of their produce remained unsold and 29.2% indicated that they had to sell their produce at below a perceived market price (Table 11). However, over all districts, 27.2% of the Bt brinjal farmers did not perceive they suffered a loss. Among the non- Bt farmers, 45.9% thought they sold their produce below a perceived market price and 6.1% reported that a large portion of their produce remained unsold. Farmer complaints about selling under a perceived market price are common across commodities.

**Table 11:** Outcomes in marketing Bt brinjal and non-Bt brinjal by district, 2019.

**Outcomes**	**Jessore**	**Tangail**	**Bogra**	**Rajshahi**	**Rangpur**	**All Districts**
**Bt brinjal**						
A large portion of the product remained unsold	36.4	37.7	4.0	32.4	0	21.0
Products sold at lower than market price of brinjal	78.8	62.5	12.0	0	0	29.2
No loss as such	15.2	27.5	2.0	97.3	0	27.2
No comments	0	2.5	4.0	0	2.9	2.1
**Non-Bt brinjal**						
A large portion of the product remained unsold	6.3	0	6.0	13.2	5.4	6.1
Products sold at lower than market price of brinjal	28.1	30.8	56.0	60.5	48.7	45.9

*Figures represent the percentage of the outcomes*.

#### Factors Influencing the Decision to Cultivate or Not Cultivate Bt Brinjal

Over all districts, 88.7% of Bt brinjal farmers chose these varieties because they believed that infestation levels of EFSB would be minimal and insecticide cost would be notably lower than if they grew non-Bt brinjal varieties (Table 12). In addition, 70.3% of Bt brinjal growers anticipated higher yields than if they grew non-Bt brinjal.

**Table 12:** Perceptions influencing the farmers' decision to grow Bt brinjal by district, 2019.

**Perceptions of Bt brinjal farmers about why they grow Bt brinjal**						
**Perception**	**Jessore**	**Tangail**	**Bogra**	**Rajshahi**	**Rangpur**	**All Districts**
Yield of Bt brinjal was higher than non-Bt brinjal	9.1	87.5	56.0	100.0	97.1	70.3
Bt brinjal was a better-quality product than non-Bt brinjal	21.2	37.5	72.0	100.0	97.1	66.2
Bt brinjal market price was higher than non-Bt brinjal	0.0	12.5	54.0	0.0	97.1	33.8
Infestation of EFSB in Bt brinjal was minimal	93.9	77.5	80.0	100.0	97.1	88.7
Insecticides costs was notably lower with Bt brinjal	93.9	97.5	76.0	100.0	97.1	91.8
**Perceptions of non-Bt brinjal farmers about why they do not grow Bt brinjal**						
**Factors**	**Jessore**	**Tangail**	**Bogra**	**Rajshahi**	**Rangpur**	**All Districts**
Lower yield of Bt brinjal than non-Bt brinjal	45.5	0	24.0	0	2.7	14.2
Lower market price of Bt brinjal than non-Bt brinjal	51.5	66.7	12.0	86.8	2.7	42.1
Almost same insecticides costs	6.1	17.9	2.0	0	0	5.1
Not getting seeds or seedlings in time	12.1	0	34.0	0	0	10.7
Not safe for human and environment	0	0	0	0	21.6	4.0
Not a suitable crop to be grown in Bangladesh	0	15.4	0	0	8.1	4.6
Didn't know about Bt brinjal	66.7	59.0	18.0	31.6	32.4	39.6

*Figures represent the percentage of the perception*.

It is interesting to note that an average of 39.6% (range of 18.0–66.7%) of the non-Bt brinjal farmers over all the districts were unaware about Bt brinjal technology. This suggests the need for focusing educational efforts on specific districts where farmers are unaware of Bt brinjal. Of those farmers who were aware of Bt brinjal (59.4% of non-Bt growers), 42.1% thought that Bt brinjal would have a lower market price than non-Bt brinjal and 14.2% thought it would have a lower yield. As the findings summarized in Table 3, Table 11 show, these perceptions appear to be incorrect.

#### Awareness of Negative Information About Bt

For both Bt and non-Bt brinjal farmers, about 80% were not aware of any negative information against Bt brinjal (Table 13). Of those who were aware of negative information (20% of the total), the main negative information they were aware of (for both Bt growers and non-Bt growers) related to the perception that Bt brinjal was not safe for human consumption or the environment. This finding should be addressed in future educational programs.

Table 13Awareness of negative information about Bt brinjal and the type of information by district.

**Table:** 

**Awareness of negative information**				
**Districts**	**Bt brinjal farmers**		**Non-Bt brinjal farmers**	
	**Aware**	**Not aware**	**Aware**	**Not aware**
Jessore	39.4	60.6	31.1	68.8
Tangail	5.0	95.0	25.6	74.4
Bogra	10.0	90.0	2.0	98.0
Rajshahi	0	100.0	0.0	100.0
Rangpur	57.1	42.9	48.6	51.4
All Districts	20.5	79.5	19.9	80.1

**Table:** 

**Type of negative information**		
**Information/Issues**	**Bt farmers**	**Non-Bt farmers**
Yield of Bt brinjal is not higher than non-Bt brinjal	7.7	4.6
Infestation of EFSB in Bt brinjal is almost the same as non-Bt brinjal	0.5	0.5
Bt brinjal is not safe for human consumption	14.4	11.2
Bt brinjal is not safe for the environment	12.3	13.3
Bt brinjal may affect surrounding crops including non- Bt brinjal	2.1	0.5
Bt brinjal is not a suitable crop to be grown in Bangladesh	0.5	0.0

*Figures represent the percentage of the farmers, 2019*.

#### Perceptions of Non-Bt Brinjal Farmers About Growing Bt Brinjal

Over three-quarters (75.5%) of the non-Bt brinjal farmers had heard opinions from neighboring farmers who were cultivating Bt brinjal (Table 14). About half (49.5%) of them heard from neighboring Bt brinjal farmers that growing Bt brinjal was a good decision. The main positive experiences heard were that Bt farmers applied less insecticides and this improved their health and environment (51.0%), and that Bt brinjal was more profitable (43.4%). This important finding should be explored with additional studies.

**Table 14:** Non-Bt farmers who heard opinions from neighboring farmers about growing Bt brinjal and what those opinions were by district.

**Were opinions heard?**						
**District**	**Jessore**	**Tangail**	**Bogra**	**Rajshahi**	**Rangpur**	**All Districts**
Heard	75.0	48.7	76.0	100.0	78.4	75.5
Did not hear	25.0	51.3	24.0	0.0	21.6	24.5
**What opinions were heard?**						
**Opinion**	**Jessore**	**Tangail**	**Bogra**	**Rajshahi**	**Rangpur**	**All Districts**
Growing Bt brinjal was a good decision	34.4	2.6	44.0	100.0	67.6	49.5
Growing Bt brinjal was a wrong/bad decision	9.4	41.0	0.00	0.0	0.00	9.7
Incurred losses compared to non-Bt farmers	34.4	41.0	0.00	0.0	0.00	13.8
Made good profit compared to non-Bt farmers	9.4	2.6	36.0	100.0	67.6	43.4
The product quality was better than non-Bt farmers	37.5	0.00	26.0	100.0	46.0	40.8
Applied less insecticide and improved farmers' health and the environment	59.4	41.0	18.0	100.0	48.7	51.0
Problem in selling and demand was less in the market	9.4	30.8	0.0	0.00	0.00	7.7
Yield of Bt brinjal was higher	0.00	0.00	24.0	84.2	24.3	27.0

*Figures represent the percentage of the farmers, 2019*.

#### Decisions by Bt and Non-Bt Farmers to Grow Bt Brinjal Next Year

When asked just after the harvest about future plans to grow Bt brinjal, 75.4% of the current year's Bt brinjal farmers and 71.4% of the current year's non-Bt farmers stated that they planned to grow Bt brinjal in the upcoming crop season (2020) (Table 15). Some Bt brinjal growers indicated that they would not plant Bt brinjal next season. The highest percentage of Bt brinjal farmers who stated they would not grow Bt brinjal next year were farmers from Jessore (48.5%) and Tangail (70.0%). These were the districts where they had concerns about selling their crop (Table 11). The non-Bt farmers who planned not to grow Bt brinjal the next season perceived there would not be high demand for Bt brinjal (8.7%) and that the low cost of insecticides would allow them to control EFSB (10.7%). Only 6.6% perceived health and environmental benefits. These findings warrant further studies on these issues.

Table 15Decision and reasons to grow Bt brinjal the following year by districts.

**Table:** 

**Decision to grow Bt brinjal**				
**Districts**	**Bt brinjal farmers**		**Non-Bt brinjal farmers**	
	**Will grow**	**Will not grow**	**Will grow**	**Will not grow**
Jessore	51.5	48.5	59.4	40.6
Tangail	30.0	70.0	10.3	89.7
Bogra	94.0	6.0	86.0	14.0
Rajshahi	100.0	0	100.0	0.0
Rangpur	97.1	2.9	97.3	2.7
All districts	75.4	24.6	71.4	28.6

**Table:** 

**Reasons expressed by the current non-Bt brinjal farmers to grow Bt brinjal the following year**						
**Reason**	**Jessore**	**Tangail**	**Bogra**	**Rajshahi**	**Rangpur**	**All Districts**
High yield of Bt brinjal	6.3	0.0	12.0	52.6	43.2	22.4
High demand in the market	0.0	0.0	8.0	2.6	32.4	8.7
No attack by EFSB	21.9	0.0	20.0	50.0	10.8	20.4
More profitable	3.1	0.0	6.0	44.7	16.2	13.8
Low cost of insecticide	15.6	0.0	18.0	0.0	18.9	10.7
Safe and good for health and environment	12.5	0.0	0.0	0.0	24.3	6.6

*Figures represent the percentage of the farmers, 2019*.

Of the current year's non-Bt farmers who planned to grow Bt brinjal next season, the main reasons for doing so were their perceptions that yield of Bt brinjal would likely be higher than non-Bt brinjal (22.4%), there would be less damage by EFSB infestation (20.4%), and higher profitability (13.8%) (Table 15). Overall, these views suggest that farmers who have grown Bt brinjal largely perceive the technology has delivered benefits, but those who have not grown Bt brinjal remain to be convinced of its potential benefits.

## Discussion

The overall objective of this research was to study the impact of Bt brinjal in the market value chain relative to locally popular non-Bt brinjal varieties, with respect to income and employment generation, and assess preference factors and perceptions about Bt brinjal that farmers had, and what they perceived the views of traders and consumers were about the product. Because of the many factors explored in this survey, analysis of each was limited to presenting results as the means of the values. Mean values provide indications of differences between the treatments and are commonly used in such agronomic surveys (e.g., Gusta et al., 2011; Hudson and Richards, 2014). However, future studies that explore many of our findings should be designed with more powerful analyses.

There were few and only minor differences in the family unit and economic status of those who chose to grow Bt brinjal or non-Bt brinjal. Thus, it appears that such socioeconomic factors did not influence the farmer's decision to grow either type of crop and analysis could justifiably focus on the crop's performance and value.

Important findings of this study indicate a 19.6% higher average yield (Table 3), a 21.7% higher average gross revenue (Table 5) and a 1.7% average revenue generation/tonne for Bt brinjal compared to non-Bt brinjal. This additional revenue/ha is equal to about $US 664, a substantial sum for resource-poor farmers in Bangladesh. This increased revenue appears to be due to higher yields and less inputs. While we did not break out pesticide costs in this study, previous reports in Bangladesh have documented a 61% decrease in pesticide costs (Rashid et al., 2018) while Ahmed et al. (2019) reported that Bt brinjal farmers spent BDT 7,174 less on pesticides/ha compared to control farmers.

Another important suggestion from this study is the variable performance of the Bt brinjal varieties in different districts relative to local varieties. This highlights that the four Bt brinjal lines are not ideally suited for all regions, not only in terms of agronomic performance but also in terms of consumer preferences relating to the fruit. This is clearly shown by the growers who chose to grow or not grow a Bt brinjal line in a particular district, such as Bt brinjal-4 which was grown in Jessore and yielded 17.6 tonnes/ha compared to 23.3 tonnes/ha in Rajshahi, a difference of 25% (Table 4).

Preference for a type of brinjal appears to be a strong consideration for consumers and marketers and includes color, shape, and size. Whether the product is Bt on not, appears to be of secondary interest, although this varies across districts. Of the farmers growing Bt brinjal, 43.1% did not face any problems selling their product, compared to 51.5% of the non-Bt brinjal farmers (Table 10). In Rangpur and Bogra, where Bt brinjal has been extensively grown since 2014, 4% of Bt farmers had a large portion of the product unsold, while in the other districts the rates were higher and the average for all regions was 21.0% (Table 11). Non-Bt brinjal farmers had an overall lower portion of their product remaining unsold. However, Bt farmers stated that 27.2% of their produce was sold at a lower perceived market price for brinjal, as compared to 45.9% of non-Bt brinjal farmers.

About 75% of non-Bt brinjal fruits were sold to wholesale traders for supply to city markets as compared to 58.2% of Bt brinjal (Table 6). Accordingly, non-Bt brinjal farmers earned 78.4% of their revenue from sales to wholesalers compared to 60.5% for Bt farmers. The primary reason for Bt brinjal remaining unsold (when this was the case) appears to be related to the non-Bt brinjal being able to better retain its skin color and texture than the four Bt brinjal varieties after 6–12 h of transportation time to the city wholesale markets. It should be noted, however, that differences in the skin and texture are related to varietal differences and are not associated with the Bt trait. As additional Bt varieties are being developed, the shipping quality and desires of the urban consumer should be considered.

Our data indicate that labor usage and wages for harvesting, grading, and packaging were higher for Bt brinjal (an additional 14.1 days of labor required and an additional BDT 730/ ha, Table 7). This reflected the higher yield of Bt brinjal, a desirable trait for a farmer. This increase in labor usage and cost was probably largely offset by the reduction in labor time and cost requirements for spraying insecticides, although this aspect of labor and insecticide costs were not specifically examined in this study.

Besides the savings in pesticide costs noted above (Rashid et al., 2018; Ahmed et al., 2019), there will be labor savings for not applying insecticides. An ex-ante study by Islam and Norton (2007) estimated that insecticide labor cost would be reduced by about $34/ha with Bt brinjal, which would offset the additional costs for harvesting and packing identified in this study. This suggests the two categories effectively cancel each other out, so the overall impact on labor usage and pesticide costs would be neutral. Additionally, the Islam and Norton study (2007) estimated that the cost of insecticides would likely be reduced by $36.36/ha. A similar pattern of labor change has been observed with the adoption of other insect-resistant crops like Bt cotton in India. For example, Qaim et al. (2006) found that reduced cotton insecticide sprayings resulted in a lower requirement for labor to undertake pest scouting and spraying (this mostly affected male family members) but this was counterbalanced by additional labor requirements for harvesting the higher yielding crop, with the latter labor change mainly affecting casual, usually female labor. Overall, they concluded that the net effect on labor use was largely neutral. Later work by Subramanian and Qaim (Subramanian and Qaim, 2009) found that the use of Bt cotton in India resulted in a net increase in labor, with the additional requirement for labor (largely female) for harvesting, outweighing the decrease in requirement for insecticide spraying.

In terms of satisfaction with the Bt brinjal technology, our data suggest that most Bt brinjal farmers were satisfied with the quality (80.6%) and yield (71.9%) of their produce (Table 9). This compared with non-Bt brinjal growers who had a similar level of satisfaction with the quality of produce (83.1%) but a lower level of satisfaction with yield (58.7%). The level of satisfaction with the technology expressed by Bt growers can also be seen in the fact that they decided to grow Bt brinjal because 88.7% believed that infestation of EFSB would be minimal and 91.8% believed their insecticide cost would be notably lower than with non-Bt brinjal (Table 12). For non-Bt growers, it is important to note that 39.6% of them had no knowledge of Bt brinjal (Table 12). Of the non-Bt growers who had some knowledge of Bt brinjal, the most important reason given for not trying Bt brinjal was fear that the fruit would obtain a lower price, followed by a view that Bt yields would be lower than non-Bt yields. Future communication efforts should focus on increasing farmers' awareness of Bt brinjal and the increased yield and revenue it generates.

In relation to the possible influence of negative information being available about Bt technology and potentially discouraging its adoption, among both Bt and non-Bt brinjal farmers a large majority (about 80% of each type of farmer) indicated that they were not aware of any negative information about Bt brinjal (Table 13). For the non-Bt brinjal growers, information about the performance of Bt brinjal appears to have had a positive influence on future planting intentions because 71.4% of the non-Bt growers indicated they would grow Bt brinjal next season (Table 14). However, this varied by district with only 10.3% in Tangail interested in growing Bt brinjal next season. The main reasons cited for future adoption of the technology was the expectation of increased yield of Bt brinjal (22.4%) and decreased attack by EFSB (20.4%). These findings suggest additional studies are warranted on these issues.

Previous studies on the impact of using Bt brinjal (see introduction) have shown virtually complete control of EFSB in Bangladesh without any disruption of non-target arthropods (Prodhan et al., 2018). In the Philippines, a similar level of control (Hautea et al., 2016) and lack of effect on non-target arthropods (Navasero et al., 2016) was observed. In both studies, the Bt lines were directly compared to their non-Bt lines (same variety but without the Bt gene) and always showed superior performance for each Bt line compared to its non-Bt line. In the present study, many comparisons were made between two brinjal groups (Bt brinjal to non-Bt brinjal), regardless of variety background. Still the trends appeared similar in that the Bt lines provided better control of EFSB, higher yields and increased revenue.

The economic benefits of Bt brinjal are reduced use and cost of insecticides, higher yields and higher returns to farmers (see for example, Rashid et al., 2018). This study is consistent with these earlier studies and extends the analysis to better understand the impacts post-farmgate and to understand the factors that influence brinjal farmers to decide to grow or not to grow Bt brinjal. The results from the present study suggest that additional follow-up studies that focus on farmers' planting decisions and consumers' purchase decisions are warranted.

The rapid adoption of the technology between 2014 and 2019 (Table 1) suggests that adopters are obtaining important benefits across a wide range of regions. In addition, as most Bt brinjal growers have not experienced difficulties in selling their produce, this suggests that the reduced levels of fruit damage make the Bt fruit more attractive to many consumers. For the future, however additional adoption of the technology will depend on availability of the Bt technology in a wider range of varieties, suitable for growing in more localities and which offer the desired characteristics of consumers. Most importantly, these findings indicate the need for an active education program for brinjal farmers since nearly 40% of them were unaware of Bt brinjal, and some had misconceptions about its safety and marketability.

## Data Availability Statement

All data generated or analyzed during this study are included in this published article.

## Author Contributions

SS, AS, MH, and VP designed the study. SS performed the research. SS and AS analyzed data. AS, SS, GB, MH, and VP wrote paper.

## Conflict of Interest

GB was employed by the company PG Economics and VP by Sathguru Management Consultants. The remaining authors declare that the research was conducted in the absence of any commercial or financial relationships that could be construed as a potential conflict of interest.
